# Plasmid-Borne and Chromosomal ESBL/AmpC Genes in *Escherichia coli* and *Klebsiella pneumoniae* in Global Food Products

**DOI:** 10.3389/fmicb.2021.592291

**Published:** 2021-02-03

**Authors:** Paula Kurittu, Banafsheh Khakipoor, Maria Aarnio, Suvi Nykäsenoja, Michael Brouwer, Anna-Liisa Myllyniemi, Elina Vatunen, Annamari Heikinheimo

**Affiliations:** ^1^Department of Food Hygiene and Environmental Health, Faculty of Veterinary Medicine, University of Helsinki, Helsinki, Finland; ^2^Finnish Food Authority, Seinäjoki, Finland; ^3^Wageningen Bioveterinary Research, Lelystad, Netherlands; ^4^Customs Laboratory, Espoo, Finland

**Keywords:** antimicrobial resistance, whole genome sequencing, extended-spectrum beta-lactamases, multidrug resistance, imported food, hybrid sequencing, one health

## Abstract

Plasmid-mediated extended-spectrum beta-lactamase (ESBL), AmpC, and carbapenemase producing Enterobacteriaceae, in particular *Escherichia coli* and *Klebsiella pneumoniae*, with potential zoonotic transmission routes, are one of the greatest threats to global health. The aim of this study was to investigate global food products as potential vehicles for ESBL/AmpC-producing bacteria and identify plasmids harboring resistance genes. We sampled 200 food products purchased from Finland capital region during fall 2018. Products originated from 35 countries from six continents and represented four food categories: vegetables (*n* = 60), fruits and berries (*n* = 50), meat (*n* = 60), and seafood (*n* = 30). Additionally, subsamples (*n* = 40) were taken from broiler meat. Samples were screened for ESBL/AmpC-producing Enterobacteriaceae and whole genome sequenced to identify resistance and virulence genes and sequence types (STs). To accurately identify plasmids harboring resistance and virulence genes, a hybrid sequence analysis combining long- and short-read sequencing was employed. Sequences were compared to previously published plasmids to identify potential epidemic plasmid types. Altogether, 14 out of 200 samples were positive for ESBL/AmpC-producing *E. coli* and/or *K. pneumoniae*. Positive samples were recovered from meat (18%; 11/60) and vegetables (5%; 3/60) but were not found from seafood or fruit. ESBL/AmpC-producing *E. coli* and/or *K. pneumoniae* was found in 90% (36/40) of broiler meat subsamples. Whole genome sequencing of selected isolates (*n* = 21) revealed a wide collection of STs, plasmid replicons, and genes conferring multidrug resistance. *bla*_CTX–M–15_-producing *K. pneumoniae* ST307 was identified in vegetable (*n* = 1) and meat (*n* = 1) samples. Successful IncFII plasmid type was recovered from vegetable and both IncFII and IncI1-Iγ types from meat samples. Hybrid sequence analysis also revealed chromosomally located beta-lactamase genes in two of the isolates and indicated similarity of food-derived plasmids to other livestock-associated sources and also to plasmids obtained from human clinical samples from various countries, such as IncI type plasmid harboring *bla*_TEM–52C_ from a human urine sample obtained in the Netherlands which was highly similar to a plasmid obtained from broiler meat in this study. Results indicate certain foods contain bacteria with multidrug resistance and pose a possible risk to public health, emphasizing the importance of surveillance and the need for further studies on epidemiology of epidemic plasmids.

## Introduction

The increasing prevalence of bacteria producing extended-spectrum beta-lactamases (ESBL) and plasmid-encoded AmpC (pAmpC) enzymes mediating resistance to many commonly used antibiotics has led to global health problems. ESBL/pAmpC are commonly found in Gram-negative enterobacteria, such as *Escherichia coli* and *Klebsiella pneumoniae*, which belong to the normal human and animal intestinal microbiota. These bacteria may spread during food production, for example during the slaughter process, and thus contaminate food products, imposing a threat to consumers.

In addition to bacterial clonal spread, certain plasmids have been shown to be successful in transferring resistance genes between bacterial populations through horizontal gene transfer, thus increasing the spread of antimicrobial resistance (AMR) (Accogli et al., 2013; Carattoli, 2013).

Typically, ESBL/AmpC-producing bacteria are not more virulent than susceptible bacteria but problems may arise when bacteria cause infections requiring treatment with antimicrobials, such as urinary tract, bloodstream, intra-abdominal, and respiratory tract infections (Pitout and Laupland, 2008). In addition to healthcare associated infections, ESBL-producing *E. coli* have been recognized as a major cause of community-onset disease (Laupland et al., 2008). The World Health Organization (WHO) has issued guidelines on the use of medically important antimicrobials in food-producing animals to preserve the effectiveness of antimicrobials important for human medicine (WHO, 2017a). Cephalosporins (third, fourth, and fifth generation) and carbapenems have been classified as critically important antimicrobials for human medicine by the WHO (WHO, 2019). The WHO has additionally published a global priority list of antibiotic-resistant bacteria to guide research, which includes specific carbapenem-resistant and third-generation cephalosporin-resistant species of the Enterobacteriaceae family, including *E. coli* and *K. pneumoniae* (WHO, 2017b).

Antimicrobial resistance can disseminate in many environments, i.e., communities, hospitals, food production plants, and farms. International travel as well as import and export of goods work as mediators of the spread of AMR across country borders. The use of antimicrobials, both quantitatively and qualitatively, varies between countries, leading to differences in AMR levels (WHO et al., 2018; EFSA and ECDC, 2020). In Europe, antimicrobial sales for food-producing animals range from 3.1 to 423.1 mg/PCU (population correction unit) (European Medicines Agency [EMA], 2019). The median for all countries participating in the report was 61.9 mg/PCU. Northern European countries use less antimicrobials in animals in general compared with Southern European countries (European Medicines Agency [EMA], 2019). A recent study by Van Boeckel et al. (2019) identified the largest hot spots of AMR in animals in China and India, with predicted hot spots emerging in Kenya and Brazil. The use of antimicrobials affects the resistance levels in food-producing animals (Hoelzer et al., 2017) and the use of certain antimicrobials in livestock has been shown to correlate with the level of AMR in *E. coli* in pigs, poultry, and cattle (Chantziaras et al., 2014). Antimicrobial use in animals can even be seen in the levels of AMR in human populations (European Centre for Disease Prevention and Control [ECDC] et al., 2017; Tang et al., 2017). As the demand for animal-source nutrition is rising with the human population increase, more antimicrobials will be used in food-producing animals, especially in low- and middle-income countries (Van Boeckel et al., 2015; Schar et al., 2018). Regarding meat products, broiler meat has been recognized as having a high prevalence of ESBL/AmpC-producing *E. coli* (Ewers et al., 2012; EFSA and ECDC, 2020).

In addition to antimicrobial use, other factors such as lack of sanitation may also affect global AMR gene diversity and abundance (Hendriksen et al., 2019). In particular, fresh food products may be susceptible to bacterial contamination from environmental sources such as poor-quality irrigation water or by cross contamination in food-producing facilities (FAO and WHO, 2019). Application of manure of animal origin is another source of AMR dissemination in agriculture and food production (Hartmann et al., 2012). In addition, seafood is often grown in developing countries in unsanitary conditions, with an increased risk of AMR (Boss et al., 2016). Subsequently, fruits and vegetables may also acquire resistant bacteria through irrigation water contaminated by aquaculture production (Done et al., 2015). Animal crops fertilized with manure and contaminated by soil bacteria may be a source of resistant bacteria for food-producing animals and lead to amplification of AMR in animal gut microbiota with concurrent antimicrobial administration (Witte, 2000; Marshall and Levy, 2011; FAO and WHO, 2019). Also, human-derived pathogenic bacteria together with AMR may enter the food chain via wastewater or sewage sludge used for irrigation or fertilization in agriculture (Reinthaler et al., 2010).

Our objective was to study the occurrence of ESBL/AmpC-producing Enterobacteriaceae in food products from a wide selection of countries and multiple food categories to assess the risk of AMR. Furthermore, to study the epidemiology of bacterial isolates and their plasmids, a subset of ESBL/AmpC-producing Enterobacteriaceae isolates were subjected to whole genome sequencing (WGS) to determine sequence types (STs), resistance and virulence genes, plasmid incompatibility (Inc) groups and subtypes, and to verify bacterial species identification. In addition, short-read sequences were combined with long-read sequences to verify beta-lactamase harboring plasmids to gain insight to plasmid epidemiology and to compare plasmid sequences to previously published plasmids to identify similarities to plasmids identified from different sources. To the best of our knowledge, this study is the first of its kind using WGS for studying AMR and beta-lactamase-harboring plasmids from a large set of import countries, with 35 countries included.

## Materials and Methods

### Sampling

Food products were collected from nine grocery stores in the Helsinki region during November 2018.

Altogether 200 individual products were collected from four different food categories, including vegetables (*n* = 60), fruits and berries (*n* = 50), meat (*n* = 60), and seafood products (*n* = 30). A detailed list of sampled products including information on country of origin, store of purchase and whether the product was fresh or frozen, and whether it originated from a same batch number with another sampled product is provided in Supplementary Table 1. Briefly, 11 batch numbers were identical for 32 sampled products. These 32 products included 10 raw broiler meat samples originating from the same batch and purchased from the same store. The other same-batch products consisted each of two or three products. Food products were divided into different categories according to how they are traditionally perceived; for example, herbs were categorized into vegetables. Products varied in size and consisted of raw, ready-to-eat, frozen, and cooked products. Country or region of origin for samples from different food categories in presented in Table 1.

**TABLE 1:** Country/region of origin for samples per food category.

		Sample category			
Country of origin	Number of samples (total)	Vegetables	Fruit and berries	Meat	Seafood
Belgium	4	3		1	
Brazil	19		12	7	
Canada	1				1
Chile	2		2		
China	3	3			
Colombia	1		1		
Costa Rica	3		3		
Denmark	4			4	
Egypt	1	1			
Estonia	1				1
European Union	1			1	
France	4	2		1	1
Germany	22	2	2	18	
Hungary	2			2	
India	1		1		
Indian Ocean	1				1
Iran	1		1		
Israel	1		1		
Italy	6	3		2	1
Kenya	2	2			
Laos	6	6			
Lithuania*	11	1		10	
Malaysia*	4	3	1		
Morocco	1		1		
Mexico	3		3		
New Zealand	3		1	2	
Norway	3				3
Pacific Ocean	9				9
Peru	5	2	3		
Poland*	4			4	
Portugal	4		4		
South Africa	5		5		
Spain	12	4	3	4	1
Thailand	6	6			
The Netherlands	3	1		2	
Turkey	1	1			
United Kingdom	1			1	
Vietnam	8		1		7
Unknown	31	20	5	1	5
**Total:**	200	60	50	60	30

*Countries of origin for products in which ESBL/AmpC-producing E. coli and/or K. pneumonia were found.

#### Subsamples

The meat category included raw broiler meat products (*n* = 10/60) from the same batch number purchased from the same store. One package of broiler meat consisted of two kilograms of raw chicken wings, and 10 of these packages were included in the study. Additional subsamples were taken from each package for further characterization of bacterial isolates to study the diversity of Enterobacteriaceae, STs, plasmid replicons, and resistance genes in raw broiler meat, which has been identified as a rich reservoir of ESBL/AmpC-producing bacteria (EFSA Panel on Biological Hazards, 2011). Four subsamples were taken from each broiler meat package, totaling 40 subsamples. The aim of subsampling was to further study the diversity of ESBL/AmpC-producing Enterobacteriaceae found in a single product.

### Country and Region of Origin of Food Samples

The country and region of origin of the samples per food category is shown in Table 1.

### Microbiological Analysis of the Samples

Samples were transported to the laboratory and stored at 4°C, and analysis was started within 24 h. Using sterile scissors and forceps, 25 g of each sample was dissected at various sites of the product and placed into a sterile Stomacher strainer bag (Seward Stomacher 400 Classic Strainer bag, Worthing, United Kingdom) with 225 ml of sterile buffered peptone water (Oxoid, Basingstoke, Hampshire, United Kingdom) and homogenized for 60 s (Stomacher 400 laboratory blender, Seward, United Kingdom). Samples were incubated at 37°C for 18–22 h. After incubation, a loopful (10 μl) of each enrichment was streaked onto two parallel MacConkey agar plates (Lab M, Lancashire, United Kingdom; Scharlau Chemie s.a, Sentmenat, Spain) with 1 mg/l cefotaxime. To improve the isolation of Enterobacteriaceae listed as the highest priority by the WHO (2017b), one of the plates was incubated at 44°C and the other at 37°C for 18–22 h. One colony from each morphologically different bacterial growth from each plate was re-streaked onto individual MacConkey agar plates with 1 mg/l cefotaxime and incubated overnight at 37°C. Bacterial colonies were re-streaked onto individual MacConkey agar plates with cefotaxime supplement until a pure culture was achieved.

### Bacterial Species Identification

Isolates were streaked onto a bovine blood agar plate and incubated at 37°C overnight for bacterial species determination with a matrix-assisted laser desorption ionization–time of flight mass spectrometry (MALDI-TOF MS) based Bruker Biotyper (Bruker Daltonics). A score value of 2.0–3.0 was considered high and thus a confident match and was set as the criteria. All isolates identified as *E. coli* and *K. pneumoniae* were stored at −70°C for further characterization.

### Antimicrobial Susceptibility Testing

Antimicrobial susceptibility testing (AST) was performed on *E. coli* and *K. pneumoniae* isolates to confirm production of ESBL, AmpC, and/or carbapenemase. AST was performed with a disk diffusion method; susceptibility to third-generation cephalosporins was tested with ceftazidime (10 μg) (Neo-Sensitabs, Rosco Diagnostica, Taastrup, Denmark) and cefotaxime (5 μg) (Oxoid, Basingstoke, Hampshire, United Kingdom), to fourth-generation cephalosporins with cefepime (30 μg), to cephamycins with cefoxitin (30 μg), and to carbapenems with meropenem (10 μg) (Neo-Sensitabs, Rosco Diagnostica, Taastrup, Denmark). Epidemiological cut-off values were used as a reference (EUCAST, 2017). Synergism between third-generation cephalosporins and clavulanic acid was tested with a combination disk diffusion test with cefotaxime + clavulanic acid (30 μg + 10 μg) and ceftazidime + clavulanic acid (30 μg + 10 μg) (Neo-Sensitabs, Rosco Diagnostica, Taastrup, Denmark). *E. coli* ATCC 25922 was included as a quality control. In addition to resistance to third-generation cephalosporins, resistance to cephamycin and <5 mm difference in inhibition zones in the combination disc diffusion test were used as criteria for AmpC production, whereas ESBL production was evidenced by resistance to third-generation cephalosporins and ≥5 mm difference in the combination disk diffusion test.

### DNA Extraction and Sequencing

#### Short-Read Sequencing

From all food samples positive for ESBL/AmpC-producing *E. coli* or *K. pneumoniae*, a collection of isolates was chosen for WGS analysis in order to study the presence of AMR, virulence genes, and plasmid replicons, as well as to assess the multilocus sequence type (MLST). If applicable, a representative from each ESBL/AmpC enzyme type category (ESBL, AmpC, or ESBL together with AmpC) and bacterial species (*E. coli* or *K. pneumoniae*) was chosen from each positive food sample, excluding subsamples. Consequently, from one to three isolates were chosen for whole genome sequencing from each positive sample.

Bacterial DNA was extracted and purified with a PureLink Genomic DNA Mini Kit (Invitrogen by Thermo Fischer Scientific, Carlsbad, CA, United States) according to the manufacturer’s instructions. The assessment of DNA quality was carried out using a NanoDrop ND-1000 spectrophotometer (Thermo Fischer Scientific, Wilmington, DE, United States) and DNA quantity was measured using a Qubit 2.0 fluorometer (Invitrogen, Life Technologies, Carlsbad, CA, United States). Library preparation was performed with an Illumina Nextera XT and sequencing with an Illumina Novaseq 6000 (Center for Genomics and Transcriptomics, Tübingen, Germany) with paired-end reads. Samples were sequenced with 100× coverage and 2× 100 bp read length.

#### Long-Read Sequencing

Subsequently, seven short-read sequenced *E. coli* isolates were chosen for long-read sequencing in order to study beta-lactamase harboring plasmids in more depth. Isolates were chosen from short-read sequenced isolates to represent a wide selection of different beta-lactamases, MLST types, and plasmid replicons. DNA extraction and purification were performed as described in Section “Short-Read Sequencing.”

DNA extracts from three or two isolates at a time were multiplexed using a SQK-LSK109 ligation sequence kit (Oxford Nanopore Technologies, United Kingdom) according to the manufacturer’s protocol. Libraries were loaded onto FLO-FLG001 R9.4.1 Flongle flow cells (Oxford Nanopore Technologies, United Kingdom) used with the MinION Mk1B sequencing device and sequenced with MinKNOW software v19.06.8 for 20–24 h.

All raw sequences have been deposited at European Nucleotide Archive (ENA) at EMBL-EBI under accession number PRJEB37779^1^. Accession numbers for each sequenced isolate are provided in Supplementary Table 2.

### Bioinformatic Analyses

#### Short-Read Sequences

Bioinformatic analyses of bacterial DNA sequences were run on a web-based service (Center for Genomic Epidemiology, DTU, Denmark). Raw reads were assembled with SPAdes 3.9 (Nurk et al., 2013) and the rest of the analyses were carried out with assembled contigs according to the service’s recommendations with default values for identity and coverage. Acquired AMR genes were determined using ResFinder 3.1 (Zankari et al., 2012), bacterial species identification was confirmed with KmerFinder 3.1 (Hasman et al., 2014; Larsen et al., 2014; Clausen et al., 2018), MLST was determined with MLST 2.0 (Larsen et al., 2012) using *E. coli* scheme 1 (Wirth et al., 2006), virulence genes for *E. coli* isolates were determined with VirulenceFinder 2.0 (Joensen et al., 2014), plasmid replicons were determined with PlasmidFinder 2.1 (Carattoli et al., 2014), and pMLST 2.0 (Carattoli et al., 2014) was used for typing plasmid replicons, where applicable.

#### Long-Read Sequences and Plasmid Analysis

Nanopore FAST5 read files were basecalled using Guppy v3.4.1 (Oxford Nanopore Technologies, United Kingdom) with FASTQ output and demultiplexed with Qcat v1.1.0 (Oxford Nanopore Technologies, United Kingdom). Quality trimming was performed with BBDuk (BBTools v38.71, Joint Genome Institute, United States) using a QTRIM value of seven. Hybrid assembly of Illumina and nanopore sequences was performed with Unicycler v0.4.8 (Wick et al., 2017) set at default values. Hybrid assembled FASTA files were uploaded to ResFinder 3.2 (Zankari et al., 2012) to determine acquired beta-lactamase resistance genes with default values for identity and coverage. PlasmidFinder 2.1 (Carattoli et al., 2014) was used to determine plasmid replicons located in the same contigs as beta-lactamase genes. Plasmid STs were determined for beta-lactamase harboring plasmid replicons with pMLST 2.0 (Carattoli et al., 2014). VirulenceFinder 2.0 (Joensen et al., 2014) was used to confirm virulence genes present on plasmid contigs. The plasmid sequences were annotated with Prokka v1.13 (Seemann, 2014) and manually curated with BLASTn/BLASTp. The newly developed tool MobileElementFinder v1.0.3 (Johansson et al., 2021) was utilized to search for mobile elements together with BLASTn/BLASTp. Plasmid structures were visualized with Easyfig v2.2.2 (Sullivan et al., 2011) for each different plasmid Inc group identified in the study, and comparisons to previously published plasmids with available metadata were visualized with BRIG v0.95 (Alikhan et al., 2011).

## Results

### Bacterial Species Identification

Altogether, 14 out of 200 food samples were positive for ESBL/AmpC-producing *E. coli* and/or *K. pneumoniae* (Table 2). ESBL/AmpC-producing *E. coli* was found in 3% (2/60) of vegetable samples. The positive samples were obtained from two coriander samples originating from Malaysia and were from the same batch and purchased from the same store. ESBL/AmpC-producing *K. pneumoniae* was found in 1% (1/60) of vegetable samples. The positive sample was obtained from chili pepper originating from Malaysia purchased from the same store as the coriander samples.

**TABLE 2:** Occurrence of ESBL/AmpC-producing Enterobacteriaceae in food samples.

Food category	Number of samples analyzed	Number of samples positive for ESBL/AmpC Enterobacteriaceae (%)^a^	Number of samples positive for ESBL/AmpC *E. coli* (%)	Number of samples positive for ESBL/AmpC *K. pneumoniae* (%)	Number of isolates obtained (samples positive for bacterial growth/total no. of samples)	Number of *E. coli* isolates/total no. of isolates (%)	Number of *K. pneumoniae* isolates/total no. of isolates (%)
Vegetables	60	3 (5)	2 (3)	1 (2)	127 (57/60)	2/127 (2)	2/127 (2)
Fruits and berries	50	0 (0)	0 (0)	0 (0)	40 (25/50)	0/40 (0)	0/40 (0)
Meat	60	11 (18)	10 (17)	2 (3)	103 (49/60)	19/103 (18)	3/103 (3)
Seafood	30	0 (0)	0 (0)	0 (0)	43 (21/30)	0/43 (0)	0/43 (0)
Total	200	14 (7)	12 (6)	3 (2)	313 (152/200)	21/313 (7)	5/313 (2)
Subsamples	40	36 (90)	35 (89)	4 (10)	135 (40/40)	86/135 (64)	4/135 (3)

*^a^Based on phenotypic tests (EFSA Panel on Biological Hazards, 2011; EUCAST, 2017) and species identification with matrix-assisted laser desorption ionization–time of flight mass spectrometry, including results with a score value of 2.0–3.0.*

**FIGURE 1:**
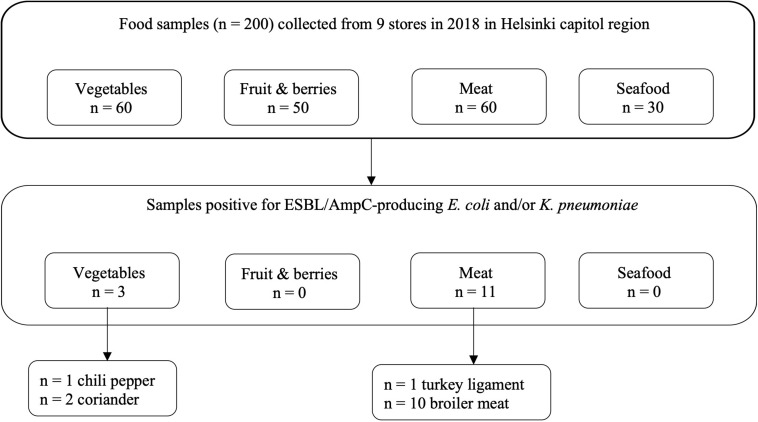
Food samples positive for ESBL/AmpC-producing *Escherichia coli* and/or *Klebsiella pneumoniae* based on phenotypic tests (EFSA Panel on Biological Hazards, 2011; EUCAST, 2017) and species identification with matrix- assisted laser desorption ionization–time of flight mass spectrometry, including results with a score value of 2.0–3.0.

ESBL/AmpC-producing *E. coli* was found in 17% (10/60) of meat samples, all originating from raw broiler meat from the same batch originating from Lithuania and purchased from the same store. In addition, ESBL/AmpC-producing *K. pneumoniae* was recovered from 3% (2/60) of meat samples, originating from the aforementioned raw broiler meat (*n* = 1) and frozen turkey meat (*n* = 1) originating from Poland. Positive samples originating from food products are presented in Figure 1.

**FIGURE 2:**
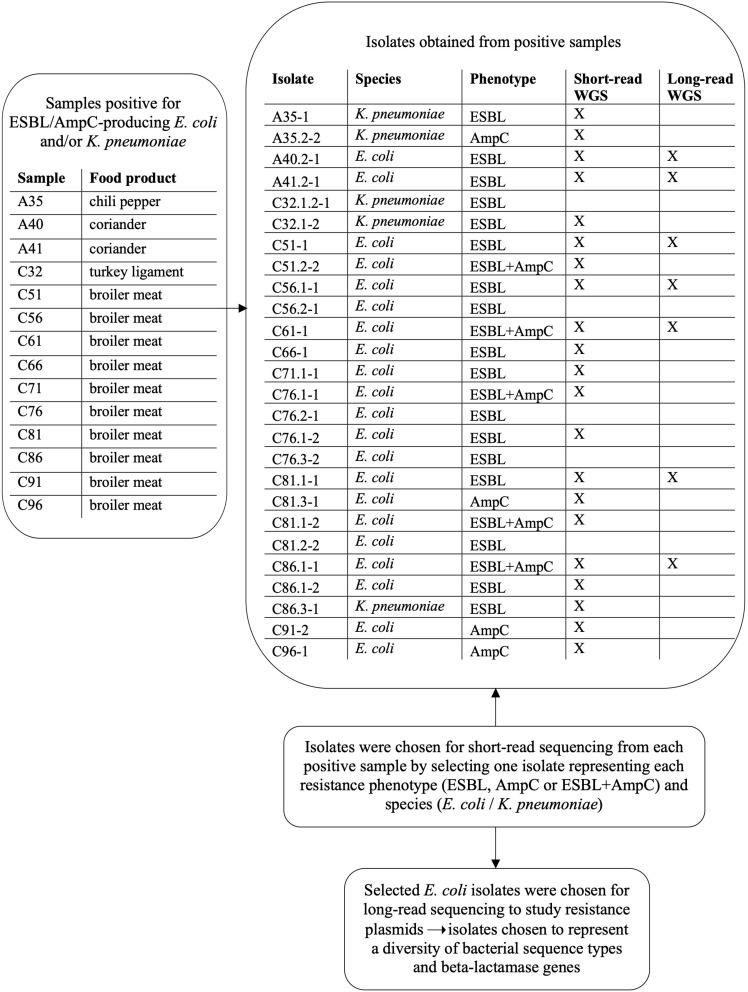
Isolates obtained from food samples positive for ESBL/AmpC-producing *Escherichia coli* and/or *Klebsiella pneumoniae* and subsequent isolate selection for short-read and long-read whole genome sequencing (WGS).

Altogether, 152 out of 200 food samples yielded a total of 313 isolates, which were subjected to bacterial species identification with MALDI-TOF MS. Isolates originated from samples incubated at both 44 and 37°C. Samples with bacterial growth (*n* = 152) yielded from one to four isolates per parallel agar plate. Information on samples and isolates positive for bacterial growth and ESBL/AmpC-producing *E. coli* and *K. pneumoniae* is provided in Figure 2. Altogether, 21 isolates were identified as *E. coli* and five as *K. pneumoniae*. Isolates positive for ESBL/AmpC-producing *E. coli* and/or *K. pneumoniae* and the selection process for WGS are presented in Figure 2.

#### Bacterial Species Identification of Subsamples

Altogether, ESBL/AmpC-producing *E. coli* and/or *K. pneumoniae* were found in 90% (36/40) of subsamples. The subsamples were taken from 10 raw broiler meat samples that were included in the main samples. These raw broiler meat samples originated all from the same batch from the same store. Altogether, 135 isolates were recovered from 40 raw broiler meat subsamples. From these subsample isolates, 109 were identified with MALDI-TOF MS with a score value of 2.0–3.0. From these isolates, 86 were identified as *E. coli* and four as *K. pneumoniae*. For 23 isolates, species identification was not possible.

### Antimicrobial Susceptibility Testing of *Escherichia coli* and *Klebsiella pneumoniae* Isolated From Food Samples

Altogether, 21 isolates from the main samples were identified as *E. coli*. From these, 13 (62%) were phenotypically ESBL producers, three (14%) AmpC producers, and five (24%) produced both AmpC and ESBL. From the five isolates from the main samples identified as *K. pneumoniae*, four were phenotypically ESBL producers and one an AmpC producer. All isolates were resistant to third-generation cephalosporin (cefotaxime and ceftazidime), except one *E. coli* isolate that was susceptible to ceftazidime but resistant to cefotaxime. None of the isolates were resistant to carbapenem (meropenem).

#### Antimicrobial Susceptibility Testing of *Escherichia coli* and *Klebsiella pneumoniae* Isolated From Raw Broiler Meat Subsamples

According to AST with disk diffusion, from the 86 isolates identified as *E. coli*, 43 (50%) were ESBL producers, 20 (23%) AmpC producers, and 23 (27%) produced both AmpC and ESBL. All four *K. pneumoniae* isolates were ESBL producers. All isolates from the subsamples were resistant to third-generation cephalosporin (cefotaxime and ceftazidime), except one *K. pneumoniae* isolate that was susceptible to ceftazidime but resistant to cefotaxime. None of the subsample isolates were resistant to carbapenem (meropenem).

### Bioinformatic Analyses

#### Short-Read Sequences

Altogether, 21 isolates were subjected to short-read WGS with Illumina, consisting of 17 *E. coli* and four *K. pneumoniae* isolates, originating from one chili pepper, two coriander, one turkey, and 10 broiler meat samples. Genotypic results are presented in Table 3.

**TABLE 3:** Genomic characteristics of *Escherichia coli* and *Klebsiella pneumoniae* strains isolated from globally produced food.

								Resistance genes									
Isolate^a^	Product	Origin	Species^b^	MLST^c^	Plasmid replicons	Phenotype^d^	Virulence genes	Aminoglycoside	Beta-lactam	Fluoroquinolone	Fosfomycin	Macrolide, Lincosamide, Streptogramin B	Phenicol	Rifampicin	Sulfonamide	Tetracycline	Trimethoprim
**A35-1**	Chili pepper	Malaysia	*K. pneumoniae*	ST307	IncFIB(K), IncFII(K)	ESBL	*N/A*	*aac(3)-IIa, aac(6′)-Ib-cr, aph(3′′)-Ib, aph(6)-Id*	*bla*_CTX–M–15_, *bla*_OXA–1_, *bla*_SHV–28_, *bla*_TEM–1B_	*aac(6′)-Ib-cr, oqxA, oqxB, qnrB1*	*fosA*		*catB3*		*sul2*	*tet*(A)	*dfrA14*
**A35.2-2**	Chili pepper	Malaysia	*K. pneumoniae*	ST101	IncFIB(K), IncFII(pK91)	AmpC	*N/A*		*bla*_DHA–1_, *bla*_SHV–28_	*oqxA, oqxB, qnrS1*	*fosA*				*sul1*	*tet*(A)	*dfrA1*
**A40.2-1**	Coriander	Malaysia	*E. coli*	ST155	IncFIB, IncFIC(FII)	ESBL	*cma, gad, IpfA*	*aph(3′)-Ia, aph(3′′)-Ib, aph(6)-Id*	*bla*_CTX–M–55_, *bla*_TEM–1B_			*mdf*(A)	*floR*	*ARR-2*	*sul2*	*tet*(A)	*dfrA14*
**A41.2-1**	Coriander	Malaysia	*E. coli*	ST479*	IncFIB, p0111	ESBL	*gad, IpfA*	*aac(3)-IV, aadA5, aph(4)-Ia*	*bla*_CTX–M–65_	*oqxA, oqxB*		*mdf*(A)	*floR*		*sul1, sul2*	*tet*(A)	*dfrA17*
**C32.1-2**	Frozen turkey ligament	Poland	*K. pneumoniae*	ST307	IncFIB(K), IncFII(K)	ESBL	*N/A*	*aac(3)-IIa, aac(6′)-Ib-cr, aph(6)-Id, aph(3′′)-Ib*	*bla*_CTX–M–15_, *bla*_OXA–1_, *bla*_SHV–28_, *bla*_TEM–1B_	*aac(6′)-Ib-cr, oqxA, oqxB, qnrB1*	*fosA*		*catB3*		*sul2*	*tet*(A)	*dfrA14*
**C51-1**	Raw broiler meat	Lithuania	*E. coli*	ST189	IncI1-I(Gamma)	ESBL	*astA, cif, eae, espA, espB, espF, espJ, gad, nleA, nleB, tir*	*aadA1, aadA2*	*bla*_SHV–12_			*mdf*(A)	*cmlA1*		*sul3*	*tet*(A)	
**C51.2-2**	Raw broiler meat	Lithuania	Escherichia fergusonii**	ST8330	ColpVC, IncB/O/K/Z, IncFIB, IncFII, IncI2, IncX1	ESBL + AmpC	*cma, gad, mchF*	*aac(3)-IV, aadA1, ant(2′′)-Ia, aph(3′)-Ia, aph(3′′)-Ib, aph(4)-Ia, aph(6)-Id*	*bla*_CMY–2_, *bla*_TEM–1B_				*catA1, floR*		*sul1, sul2*	*tet*(B)	
**C56.1-1**	Raw broiler meat	Lithuania	*E. coli*	ST4994	Col156, Col8282, ColpVC, IncI1-I(Gamma), IncI2(Delta)	ESBL	*air, astA, celb, eilA, gad, iha, iss*		*bla*_TEM–52C_			*mdf*(A)					
**C61-1**	Raw broiler meat	Lithuania	*E. coli*	ST1011	IncFIB, IncFII, IncI1-I(Gamma)	ESBL + AmpC	*cma, eilA, iroN, iss, mchF*	*aadA2, aph(3′′)-Ib, aph(6)-Id*	*bla*_CMY–2_, *bla*_TEM–1B_			*mdf*(A)			*sul2*	*tet*(A)	*dfrA12*
**C66-1**	Raw broiler meat	Lithuania	*E. coli*	ST423	IncFIB(pLF82), IncFII(pSE11), IncI1-I(Gamma)	ESBL	*gad, IpfA*	*aadA1*, *aadA2*	*bla*_CARB–2_, *bla*_SHV–12_			*mdf*(A)	*cmlA1*		*sul3*	*tet*(A)	*dfrA16*
**C71.1-1**	Raw broiler meat	Lithuania	*E. coli*	ST1485	IncB/O/K/Z, IncFIA, IncFIB, IncFIC(FII), IncHI2, IncHI2A, p0111	ESBL	*air, eilA, gad, iha, iroN, IpfA, iss, mchF, mcmA, tsh*	*aadA1, aadA2, aph(3′′)-Ib, aph(6)-Id*	*bla*_CMY–2_, *bla*_TEM–1B_			*mdf*(A)	*cmlA1*		*sul2, sul3*	*tet*(A)	*dfrA14*
**C76.1-1**	Raw broiler meat	Lithuania	*E. coli*	ST201	IncFIB, IncFII(pCoo), IncI1-I(Gamma)	ESBL + AmpC	*cma, gad, iroN, IpfA, iss*	*aadA1*	*bla*_CMY–2_, *bla*_TEM–1B_	*qnrS1*		*mdf*(A)	*cmlA1*		*sul3*	*tet*(A)	*dfrA15*
**C76.1-2**	Raw broiler meat	Lithuania	*E. coli*	ST83*	IncFIA, IncFIB, IncI1-I(Gamma)	ESBL	*astA, gad, iroN, iss, mchF, tsh*	*aadA1, aadA2*	*bla*_SHV–12_			*mdf*(A)	*cmlA1*		*sul3*	*tet*(A)	
**C81.1-1**	Raw broiler meat	Lithuania	*E. coli*	ST38	Col156, IncFII(29), IncI1-I(Gamma)	ESBL	*air, eilA, gad*	*aadA5*	*bla*_CTX–M–1_			*mdf*(A)			*sul2*		*dfrA17*
**C81.3-1**	Raw broiler meat	Lithuania	*E. coli*	ST1638	IncFIB, IncFIC(FII), IncFII(pHN7A8), IncI1-I(Gamma), IncX1, Col(pHAD28)	AmpC	*gad, iss*	*aadA1, aadA2, aph(3′′)-Ib, aph(6)-Id*	*bla*_CMY–2_, *bla*_TEM–1B_	*qnrB19*		*mdf*(A)	*cmlA1*		*sul3*	*tet*(B)	*dfrA8*
**C81.1-2**	Raw broiler meat	Lithuania	*E. coli*	ST641	IncFIB, IncFIB(pLF82), IncFII(pSE11), IncX1	ESBL + AmpC	*etpD, gad, IpfA, iss*	*aadA1, aadA2*	*bla*_CARB–2_, *bla*_CMY–2_, *bla*_TEM–1B_	*qnrS1*		*mdf*(A)	*cmlA1*		*sul3*	*tet*(A)	*dfrA16*
**C86.1-1**	Raw broiler meat	Lithuania	*E. coli*	ST641	IncFIB, IncFIB(pLF82), IncFII(29), IncFII(pSE11), IncX1	ESBL + AmpC	*etpD, gad, iss, IpfA*	*aadA1, aadA3*	*bla*_CARB–2_, *bla*_CMY–2_, *bla*_TEM–1B_	*qnrS1*		*mdf*(A)	*cmlA1*		*sul3*	*tet*(A)	*dfrA16*
**C86.1-2**	Raw broiler meat	Lithuania	*E. coli*	ST38	Col156, IncFII(29), IncI1-I(Gamma)	ESBL	*air, eilA, gad, ireA*	*aadA1, aadA5, aph(3′′)-Ib, aph(6)-Id*	*bla*_CTX–M–1_			*mdf*(A)	*floR*		*sul1, sul2*	*tet*(A)	*dfrA17*
**C86.3-1**	Raw broiler meat	Lithuania	*K. pneumoniae*	ST37	IncFIA(HI1), IncR	ESBL	*N/A*	*aac(3)-IId, aph(3′′)-Ib, aph(6)-Id*	*bla*_CTX–M–15_, *bla*_SHV–81_, *bla*_TEM–1B_	*oqxA, oqxB*	*fosA*				*sul2*		*dfrA14*
**C91-2**	Raw broiler meat	Lithuania	*E. coli*	ST117	ColpVC, IncB/O/K/Z, IncFIB, IncFIC(FII), IncFII(29)	AmpC	*celb, gad, ireA, iroN, IpfA, iss, mchB, mchC, mchF, pic*	*aadA1, aph(3′′)-Ib, aph(6)-Id*	*bla*_CMY–2_, *bla*_TEM–1B_			*mdf*(A)			*sul1, sul2*	*tet*(A)	*dfrA8*
**C96-1**	Raw broiler meat	Lithuania	*E. coli*	ST88	IncFII(29), IncI1-I(Gamma)	AmpC	*gad, iss, IpfA*	*aadA1, aph(3′′)-Ib, aph(6)-Id*	*bla*_CMY–2_, *bla*_TEM–1B_			*mdf*(A), *mph*(B)	*catA1*		*sul1, sul2*		*dfrA1, dfrA8*

^a^Suffix −1 indicates first incubation temperature of 44°C and −2 of 37 °C. ^b^Analysis tools by Center for Genomic Epidemiology: SPAdes 3.9 for assembly, KmerFinder 3.1 for species determination (** identified as E. coli with SpeciesFinder 1.2), MLST 2.0 for multilocus sequence typing [* scheme Escherichia coli #1 used first, if sequence type (ST) unidentified then #2 (Jaureguy et al., 2008) used], PlasmidFinder 2.0 for plasmid replicons, VirulenceFinder 2.0 for E. coli virulence genes (N/A, not applicable for K. pneumoniae), ResFinder 3.1 for acquired resistance genes. ^c^MLST, multilocus sequence typing. ^d^Based on phenotypic tests; ESBL, extended-spectrum beta-lactamase.

The sequenced isolates were confirmed to be either *E. coli* or *K. pneumoniae*. Altogether 18 MLST types were identified, 15 STs from the 17 sequenced *E. coli* isolates and three STs from the four sequenced *K. pneumoniae* isolates. Four different STs were identified from the four vegetable samples: ST155 and ST479 *E. coli* isolates from coriander, and ST307 and ST101 *K. pneumoniae* isolates from chili. The *K. pneumoniae* isolate from turkey meat was identified as ST307. The 16 isolates originating from broiler meat consisted of 14 different STs, including ST189, ST8330, ST4994, ST1011, ST423, ST1485, ST201, ST83, ST38, ST1638, ST641, ST117, and ST88 *E. coli* isolates and ST37 *K. pneumoniae* isolate.

Multiple AMR genes were observed in all of the sequenced isolates. All isolates harbored beta-lactamase gene(s) (*bla*_CTX–M–1,_ *bla*_CTX–M–15_, *bla*_CTX–M–55_, *bla*_CTX–M–65_, *bla*_SHV–12_, *bla*_SHV–81_, *bla*_SHV–28_, *bla*_TEM–1B_, *bla*_TEM–52C_, *bla*_CARB–2_, *bla*_OXA–1_, *bla*_DHA–1_, and/or *bla*_CMY–2_) and almost all isolates harbored genes conferring resistance to aminoglycoside, macrolide, lincosamide, streptogramin B, phenicol, sulfonamide, tetracycline, and trimethoprim. Additionally, a few isolates harbored plasmid-mediated quinolone resistance (PMQR), fosfomycin, and/or rifampicin resistance genes. Nine out of the 16 sequenced broiler meat samples harbored the AmpC beta-lactamase *bla*_CMY–2_.

All isolates were carrying AMR genes conferring resistance toward critically important antimicrobials (WHO, 2019), including aminoglycosides, third- and fourth-generation cephalosporins, macrolides, and quinolones. All of the *E. coli* isolates harbored the macrolide resistance gene *mdf(A).* All of the *K. pneumoniae* isolates harbored PMQR and fosfomycin resistance genes, whereas only five *E. coli* isolates harbored PMQR genes and none harbored fosfomycin resistance genes. Aminoglycoside resistance genes were found in all but one *E. coli* and one *K. pneumoniae* isolate. Two *K. pneumoniae* isolates harbored gene *aac(6′)-Ib-cr*, which confers resistance toward both aminoglycosides and fluoroquinolones (Frasson et al., 2011). Phenicol resistance was found in 16 isolates, rifampicin resistance in one isolate, sulfonamide resistance in all but one isolate, and tetracycline and trimethoprim resistance in 17 isolates.

ESBL/AmpC phenotypes correlated with the detected genotype in all but four (isolates A35.2-2, C51.2-2, C61-1, and C71.1-1) of the sequenced isolates. Isolate A35.2-2 was phenotypically an AmpC producer but harbored both *bla*_DHA–1_ and *bla*_SHV–28_. Isolates C51.2-2 and C61-1 were phenotypically both ESBL and AmpC producers, although they possessed only *bla*_TEM–1B_ in addition to *bla*_CMY–2_. Isolate C71.1-1 was phenotypically an ESBL producer but harbored *bla*_CMY–2_ in addition to *bla*_TEM–1B_. *bla*_TEM–1B_ confers resistance toward penicillin and ampicillin but not significantly toward extended-spectrum cephalosporins (Paterson and Bonomo, 2005). Complete resistance gene profiles are shown in Table 3.

A total of 24 different plasmid replicons were identified from the sequenced isolates with PlasmidFinder 2.1 (Carattoli et al., 2014) with IncF subtype IncFIB and IncI1-Iγ being the most common and appearing in 11 and 10 isolates, respectively. Plasmid replicons belonging to IncF groups were overall detected in 19 isolates. Each isolate harbored from one to seven different plasmid replicons, with the average amount of replicons being three per isolate. Typing of plasmids with plasmid multilocus sequence typing (pMLST) revealed 14 different STs, with the most common ones being ST12 and ST95-CC9 (clonal complex) appearing each in three isolates. Three ST/clonal complex clusters were linked to the IncI1-Iγ plasmid group. IncI1-Iγ ST3-CC3 was found in two ST38 *E. coli* isolates, both harboring resistance gene *bla*_CTX–M–1_. IncI1-Iγ ST95-CC9 was associated with resistance gene *bla*_SHV–12_, which was found in three *E. coli* isolates, all with different STs: ST189, ST423, and ST83. IncI1-Iγ ST12 was identified in three *E. coli* isolates harboring *bla*_CMY–2_. All of the IncI1-Iγ plasmid replicons originated from broiler meat samples originating from the same batch and collected from the same store. pMLST results are presented in Supplementary Table 3.

The isolates harbored multiple different virulence genes. Virulence gene results are presented in Table 3. One of the isolates, originating from broiler meat, was positive for adhesin intimin coding *eae* gene, which is associated with enteropathogenic *E. coli* (Frankel et al., 1998; Müller et al., 2016).

#### Plasmid Analysis

Altogether, seven *E. coli* isolates were subjected to long-read sequencing with Oxford Nanopore Technologies, consisting of two coriander and five broiler meat isolates. Results of the hybrid assembled plasmid sequences are presented in Table 4. All long-read sequenced and hybrid assembled isolates were found to harbor resistance genes and plasmid replicons matching with short-read sequence analysis. In the two coriander isolates, beta-lactamase genes were located in plasmid replicons belonging to the IncF group, whereas in four out of five broiler isolates beta-lactamase genes were located on IncI1-Iγ plasmids. Isolate C86.1-1 included *bla*_CARB–2_-carrying IncF type plasmid replicon and *bla*_TEM–1_-carrying IncX1 plasmid replicon in addition to a chromosomal *bla*_CMY–2_. A chromosomal *bla*_TEM–1B_ gene was detected also in isolate C61-1. All detected plasmid replicons represented different plasmid STs. Additionally, all IncF plasmid replicons, the IncX1 replicon and one IncI1-Iγ replicon were found to harbor multiple resistance genes.

**TABLE 4:** Hybrid assembled *Escherichia coli* food isolates and corresponding beta-lactamase harboring plasmids for comparative analysis.

Isolate	ESBL-plasmid	Plasmid replicon^a^	pMLST/RST	Plasmid size (bp)	Beta-lactamase gene	Other resistance genes on same plasmid replicon(s)	Virulence genes on plasmid replicon(s)
**A40.2-1**	pZPK-A40.2-1	IncFIB, IncFIC(FII)	[F-:A-:B1] FIC-4	120698	*bla*_CTX–M–55,_ *bla*_TEM–1B_	*ARR-2, floR, dfrA14, aph(3′′)-Ib, aph(3′)-Ia, aph(6)-Id, tet(A), sul2*	*cma, cvaC, hlyF, iucC, iutA, ompT, sitA*
**A41.2-1**	pZPK-A41.2-1	IncFIB, p0111	[F-:A-:B76]	153291	*bla*_CTX–M–65_	*floR, oqxA, oqxB, dfrA17, aac(3)-IV, aadA5, aph(4)-Ia, sul1, sul2*	*papC*
**C51-1**	pZPK-C51-1	IncI1-Iγ	95 (CC-9)	121837	*bla*_SHV–12_	*aadA1, aadA2b, tet(A), sul3, cmlA1*	*cib*
**C56.1-1**	pZPK-C56.1-1	IncI1-Iγ	36 (CC-3)	89504	*bla*_TEM–52C_	–	–
**C61-1**	pZPK-C61-1	IncI1-Iγ	2 (CC-2)	97275	*bla*_CMY–2_	–	*cia*
**C81.1-1**	pZPK-C81.1-1	IncI1-Iγ	3 (CC-3)	112374	*bla*_CTX–M–1_	–	*cib*
**C86.1-1**	pZPK-C86.1-1_X1	IncX1	–	47686	*bla*_TEM–1B_	*qnrS1*	

*^a^Analysis tools by Center for Genomic Epidemiology: ResFinder 3.2 for acquired resistance genes; PlasmidFinder 2.1 for determining beta-lactamase harboring plasmid replicons; pMLST 2.0 for plasmid sequence type (ST) (CC, clonal complex; pMLST, plasmid multilocus sequence typing; RST, replicon sequence typing).*

##### Comparison of IncF type plasmids

Three of the hybrid assembled food isolates were found to carry plasmids belonging to the IncF group. Two of the isolates were of coriander origin (A40.2-1 and A41.2-1) and one from raw broiler meat (C86.1-1). RST analysis with pMLST tool (Carattoli et al., 2014) indicated new FIB alleles for pZPK-A41.2-1 and pZPK-C86.1-1 which were submitted to PubMLST database^2^ and assigned FIB76 and FIB77, respectively. IncFII/IncFIB replicon carrying narrow-spectrum beta-lactamase *bla*_CARB–2_ with a FAB formula of F68:A-:B77 from isolate C86.1-1 additionally carried *bla*_CMY–2_ in the chromosome and an IncX1 plasmid harboring *bla*_TEM–1_. From this isolate, the IncX1 plasmid (pZPK-C86.1-1_X1) was selected for further comparison analysis to achieve diverse ESBL-plasmid comparisons (Table 4).

**FIGURE 3:**
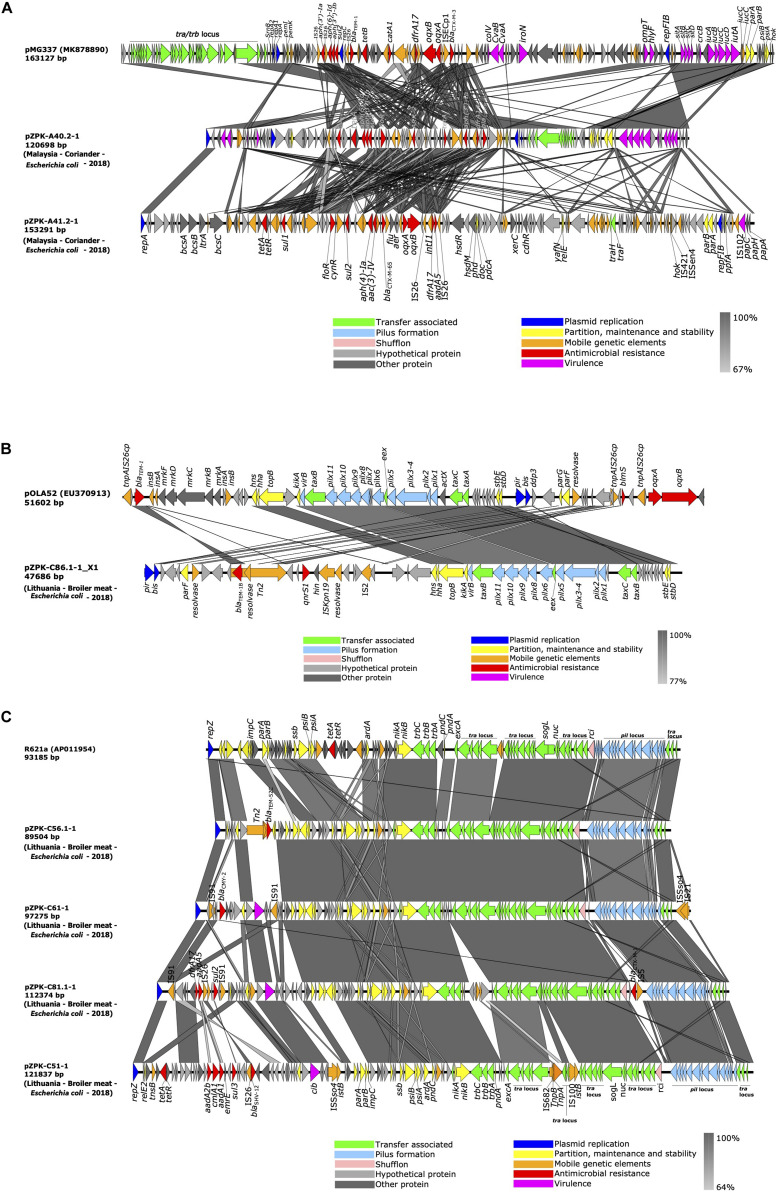
Linear comparison of **(A)** IncFIB/IncFIC(FII) and IncFIB/p0111, **(B)** IncX1 and **(C)** IncI1-Iγ plasmids identified in this study with previously published plasmids (GenBank accession numbers in parentheses for reference plasmids; for plasmids identified in this study the country of origin, source, bacterial species and year of isolation is provided in parentheses). Gray areas between plasmid sequences indicate the percentage of nucleotide sequence identity. The arrows represent coding sequences and their orientation and are colored based on their predicted function.

pZPK40.2-1 from isolate A40.2-1 carried a multireplicon IncFIB/IncFIC with a size of 120.7 kb and C + G content of 51.45% and 136 predicted coding sequences (CDSs). pZPK41.2-1 from isolate A41.2-1 carried IncFIB replicon together with p0111 and was 153.3 kb in size with G + C content of 49.93% and 165 CDSs. Comparison to a previously published and annotated IncF plasmid with FAB formula F18:A-:B1:C4 (GenBank accession: MK878890.1) indicated high variability between the plasmids with multiple insertions of mobile elements in both pZPK-A40.2-1 and pZPK-A41.2-1 (Figure 3A).

**FIGURE 4:**
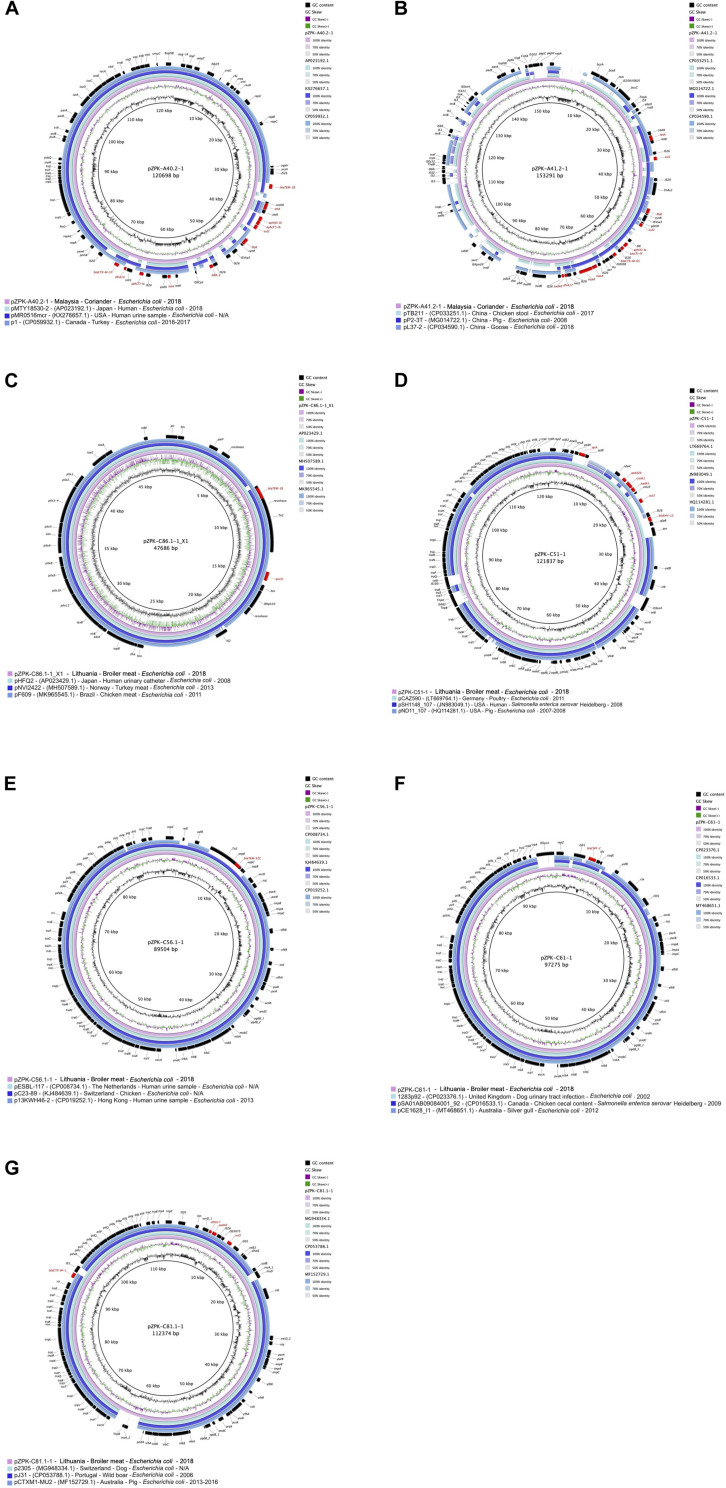
Circular comparisons of the studied plasmids to previously published similar plasmids (GenBank accession numbers are provided in parentheses in the figure legends after plasmid name, followed by country, source, bacterial species and year of isolation; N/A, not available). GC content and GC skew of the studied plasmids are depicted in the inner map with distance scale and the outer ring represents predicted coding sequences with antimicrobial resistance genes highlighted in red. The plasmids in this study included **(A)** pZPK-A40.2-1, **(B)** pZPK-A41.2-1, **(C)** pZPK-C86.1-1_X1, **(D)** pZPK-C51-1, **(E)** pZPK-C56.1-1, **(F)** pZPK-C61-1 and **(G)** pZPK-C81.1-1.

pZPK-A40.2-1 harbored multiple resistance genes in addition to its beta-lactamases *bla*_CTX–M–55_ and *bla*_TEM–1B_, including *ARR-2, floR, dfrA14, aph(3′′)-Ib, aph(3′)-Ia, aph(6)-Id, tet(A)* and *sul2*. In addition, multiple virulence genes (*cma, cvaC, hlyF, iucC, iutA, ompT, sitA*) were detected. Top BLAST hits with available metadata in NCBI included an IncFII/IncFIC type plasmid with a FAB profile F18:A-B1 and *bla*_CTX–M–55_, *bla*_TEM–1B_ and *mcr-1* from a human urine sample from United States (GenBank accession: KX276657.1) with 89% coverage and 99.73% identity. Another plasmid with the FAB profile F18:A-B1 with *bla*_CTX–M–55_ obtained from a turkey in Canada (GenBank accession: CP059932.1) was found to be similar with 88% coverage and 99.78% identity (Figure 4A).

pZPK-A41.2-1 also carried multiple resistance genes in addition to the beta-lactamase *bla*_CTX–M–65_, including *dfrA17, oqxB, oqxA, sul2, sul1, aac(3)-IV, aph(4)-Ia, aadA5, tet(A)* and *floR*. The only virulence gene detected was the P fimbriae encoding *papC*. BLASTn search against NCBI database identified only partly similar plasmid sequences with lower coverage values than for the other plasmids in the study. pZPK-A41.2-1 aligned with 68% coverage and 99.97% identity with another IncFIB/p0111 type plasmid obtained from chicken stool in China (GenBank accession: CP033251.1). This plasmid carried a *bla*_CTX–M–14_ instead of *bla*_CTX–M–65_. Another IncFIB/p0111 plasmid with *bla*_TEM–1B_ from China recovered from a goose sample (GenBank accession: CP034590.1) aligned with 64% coverage and 99.5% identity (Figure 4B).

##### Comparison of IncX1 type plasmid

IncX1 type plasmid (pZPK-C86.1-1_X1) was recovered from isolate C86.1-1 from raw broiler meat. The plasmid carried *bla*_TEM–1B_ and PMQR gene *qnrS1* and no virulence genes were identified. The size of the plasmid was 47.7 kb with G + C content 43.13% and 56 CDSs predicted. Comparison of the plasmid structure to a IncX1 reference plasmid pOLA52 (GenBank accession: EU370913.1) identified the IncX plasmid backbone in the sequenced plasmid, including replication genes *pir* and *bis*, pilus associated genes *pilX* and genes involved in partition (*par*), stability (*stb*) and conjugation (t*ax*) (Johnson et al., 2012) (Figure 3B).

BLASTn search against NCBI database identified a highly similar plasmid obtained from *E. coli* from a human urinary catheter sample in Japan (GenBank accession: AP023429.1), aligning with 100% coverage and 99.98% identity and also carrying resistance genes *bla*_TEM–1B_ and *qnrS1*. Another highly homologous plasmid with the same resistance profile was a plasmid obtained from turkey meat in Norway (GenBank accession: MH507589.1), aligning with 99% coverage and 99.99% identity. An IncX1 plasmid carrying *bla*_CTX–M–15_, *bla*_TEM–1B_ and *qnrS1* recovered from chicken meat in Brazil (GenBank accession: MK965545.1) was also found to be similar, with 97% coverage and 100% identity to pZPK-C86.1-1_X1 (Figure 4C).

##### Comparison of IncI1-Iγ type plasmids

IncI1-Iγ type plasmid replicons were identified from four hybrid sequenced food isolates, all originating from broiler meat from the same batch and ranging in size from 89.5 to 121.8 kb. Overall G + C content ranged in size from 50.24 to 51.65% and between 97 and 136 CDSs were predicted per plasmid. Only one of the four IncI1-Iγ plasmids harbored additional resistance genes other than a beta-lactamase; this pZPK-C51-1 *bla*_SHV–12_-harboring ST95(CC-9) plasmid with *aadA1, aadA2b, tet(A), sul3, cmlA1* was the largest of the four plasmids. The only virulence genes detected in IncI1-Iγ plasmids were the channel-forming colicin gene *cia* or *cib*. Plasmid pZPK-C56.1-1 (ST36 and CC-3) carried *bla*_TEM–52C_ and no virulence genes. The *pndCA* plasmid addiction system was detected in all the IncI1-Iγ plasmid sequences.

Aligning the plasmid sequences with BLASTn with both IncI1 type reference plasmid R64 (GenBank accession: AP005147.1) and IncIγ type reference plasmid R621a (GenBank accession: AP011954.1) indicated three of the four of the plasmids were slightly more similar to the IncIγ type R621a, which was chosen for comparative genomic visualization (Figure 3C). The plasmids in our study aligned with a query coverage of 61–85% and with >98% identity with R621a. All studied plasmids had typical IncI type backbones including the conjugational, pilus formation and maintenance and stability regions, except for *bla*_CTX–M–1_ and IS*5* located near the shufflon region in pZPK-C81.1-1. The accessory module was variable between plasmids with different inserted elements and resistance genes (Figure 3C). Alignment of *excA* and *traY* regions with both references (R64 and R621a) indicated more similarity with IncI1 type R64. The *parAB* region was also more similar to corresponding regions of R64 in all sequenced IncI1-Iγ plasmids except for pZPK-C51-1 which shared more similarity with *parAB* region of R621 plasmid. The results indicate the plasmids in our study share similarities with both I1 and Iγ replicon types for which reason plasmid results are here referred to as IncI1-Iγ.

BLASTn search against NCBI database indicated highly similar plasmids have been isolated from different locations and sources, mostly from different livestock but also human clinical samples and wild animal sources (Figures 4D–G). pZPK-C56.1-1 was found to be highly similar with a plasmid of the same ST type (ST36) also carrying *bla*_TEM–52C_ isolated from a human urine sample in the Netherlands (GenBank accession: CP008734.1) with a coverage of 100% and identity of 99.99%. pZPK-C56.1-1 was found to align also with another plasmid from a human urine sample from Hong Kong (GenBank accession: CP019252.1) with 91% coverage and 98.98% identity. Also, pZPK-C51-1 carrying *bla*_SHV–12_ matched with 99.76% identity and 80% coverage to a plasmid isolated from *Salmonella enterica* serovar Heidelberg from a human source in United States (GenBank accession: JN983049.1). *bla*_CMY–_2-harboring pZPK-C61-1 was similar with a plasmid with the same sequence type ST2 CC-2 obtained from a dog urinary tract infection sample in the United Kingdom (GenBank accession: CP023376.1), aligning with 96% coverage and 99.99% identity. pZPK-C61-1 was also similar to a plasmid isolated from a silver gull sample from Australia (GenBank accession: MT468651.1) with 93% coverage and 98.65% identity. Also, pZPK-C81.1-1 with *bla*_CTX–M–1_ matched with a plasmid obtained from a dog source in Switzerland (GenBank accession: MG948334.1) with 96% coverage and 99.92% identity and with a wild animal derived plasmid from a wild boar in Portugal (GenBank accession: CP053788.1) with 94% coverage and 99.98% identity. All IncI1-Iγ type plasmids in this study were found to match with similar plasmids from livestock-associated sources, such as a ST95 plasmid with *bla*_SHV–12_ from poultry in Germany (GenBank accession: LT669764.1) with 94% coverage and 99.97% identity with pZPK-C51-1 and ST36 plasmid with *bla*_TEM–52C_ from chicken in Switzerland (GenBank accession: KJ484639.1) with 100% coverage and 99.96% identity with pZPK-C56. 1-1.

## Discussion

Our results demonstrate that imported food products from various origins, acquired in Finland, possess wide genetic variety of ESBL/AmpC-producing Enterobacteriaceae. Hybrid sequence analysis combining long- and short-read sequencing proved beneficial in determining resistance gene locations on specific plasmid replicons. Hybrid assembly of plasmid sequences also resolved the chromosomal location of beta-lactamase genes in two studied isolates. The spread of ESBL/pAmpC genes is highly attributed to epidemic and highly transmissible plasmids, which emphasizes the importance of plasmid replicon determination in epidemiological studies. Although the sample size was limited, our study provides a global indicative insight into AMR prevalence in global food products from 35 countries. The diversity of AMR genes was high in the isolates from food samples positive for ESBL/AmpC-producing Enterobacteriaceae. In addition, resistance toward critically important antimicrobials was found in all sequenced isolates. Raw meat, in particular broiler meat, was recognized as a common source of ESBL/AmpC-producing Enterobacteriaceae. Broiler meat samples originated from the same batch, which emphasizes the finding that one source can contain a wide variety of different resistance genes, STs, and plasmid replicons.

Compared with an earlier study investigating ESBL/AmpC-producing *E. coli* in Finnish poultry production, the isolates recovered from broiler meat in the current study showed broader resistance to multiple antimicrobial groups (Oikarainen et al., 2019). The isolates in the present study carried AMR genes conferring resistance against critically important antimicrobials (WHO, 2019), including aminoglycosides, third- and fourth-generation cephalosporins, macrolides, and quinolones, whereas no macrolide or PMQR was found in the isolates recovered from Finnish poultry production in the earlier study (Oikarainen et al., 2019). Another study of broilers at slaughterhouses found only limited sulfonamide resistance in addition to ESBL/AmpC with no other resistance genes in broiler meat and cecum samples (Päivärinta et al., 2020).

Our results show that vegetables, coriander and chili from Malaysia contained ESBL/AmpC-producing bacteria. Vegetables are often eaten without heating processes, leading to a greater risk of acquiring resistant bacteria. The three vegetable samples positive for ESBL/AmpC-producing Enterobacteriaceae in our study yielded a total of four isolates with varying STs, plasmid replicons, and a wide variety of beta-lactamase encoding genes, including *bla*_CTX–M–15_, *bla*_SHV–__28_, *bla*_CTX–M–55_, and *bla*_CTX–M–65_. Samples from all food categories yielded many isolates, although most isolates were recovered from the vegetable samples. *Citrobacter* spp. and *Hafnia alvei* were also identified in the isolates, but these were left out of further analysis as our study focused on *E. coli* and *K. pneumoniae*, which are categorized as critical for research by the WHO (WHO, 2017b).

The finding of resistance genes *bla*_CTX–M–15_, *bla*_SHV–12_, and *bla*_OXA–1_ commonly linked to human sources (Cantón et al., 2008; Livermore et al., 2019) highlights the potential transmission route of AMR via food products and emphasizes the importance of hygiene measures. In the current study, two of the three isolates carrying *bla*_CTX–M–15_ were harboring the plasmid replicon IncFII(K), which has been previously identified from human infections with *K. pneumoniae* harboring *bla*_CTX–M–15_ (Dolejska et al., 2012; Bi et al., 2018). Plasmids of the IncFII type have been identified as being epidemic (Carattoli, 2009; Mathers et al., 2015). The AmpC-type resistance gene *bla*_CMY–2_ and ESBL types *bla*_CTX–M–1_, *bla*_CTX–M–55_, and *bla*_CTX–M–65_ are associated with food-producing animals, especially poultry (EFSA Panel on Biological Hazards, 2011; Cormier et al., 2019; Day et al., 2019). Interestingly, *bla*_CMY–2_ and *bla*_CTX–M–1_ were recovered from broiler meat samples and *bla*_CTX–M–55_, and *bla*_CTX–M–65_ from vegetable samples in our study. One explanation for the finding of AMR genes associated with food-producing animals in food products could be the possible use of contaminated irrigation water or animal manure in the farming process. As low-income countries have higher AMR abundance in their wastewater (Hendriksen et al., 2019), it can be speculated that the risk of ESBL/AmpC dissemination is greater, especially when water is contaminated by animal manure or wastewater from human sources is used for crop watering. However, no conclusions can be drawn from the current study regarding the possible differences in ESBL/AmpC-producing Enterobacteriaceae prevalence between different countries, because the sample size per country was limited. More attention should be paid to the comparison of different food categories from the results of the current study.

Food samples were found to harbor a variety of bacterial STs, some of which have been described from human infections, i.e., ST37 from Chinese strains (Zhu et al., 2015; Zhang et al., 2016; Xiao et al., 2017) and ST307 from Italian, Colombian, and United Kingdom strains (Villa et al., 2017) of *K. pneumoniae* and ST38 from Bangladeshi and United Kingdom strains of *E. coli* (Hasan et al., 2015; Day et al., 2019). Also, same ST types (ST88 and ST117) found in the Lithuanian broiler meat in the present study have been previously found also in Finnish broiler production (Heikinheimo et al., 2016; Oikarainen et al., 2019) and ST117 more widely in Nordic broiler production (Ronco et al., 2017). In a Canadian study (Vincent et al., 2010), *E. coli* ST117 isolates were identified from human infections and poultry sources with related pulsed-field gel electrophoresis profiles, pointing to a possible poultry source of human infections. With the large variety of different bacterial STs, it is difficult to draw any further conclusions about possible bacterial transmission routes from our study, but it seems that certain *E. coli* STs and their resistance genes are common in international food production and are found in food products produced in various countries. Noteworthy is the finding of clinically relevant bacterial STs.

Plasmid replicons linked to the spread of AMR, particularly of the IncI type (Carattoli, 2013), were detected in our study. IncI1-Iγ ST12 was recovered from three broiler isolates, and it has been previously linked to *bla*_SHV–12_ from avian *E. coli* (Accogli et al., 2013). In addition, IncI plasmids with the *bla*_CTX–M–1_ gene have been found in *E. coli* from poultry and humans in the Netherlands (Leverstein-van Hall et al., 2011). Also, plasmids of the IncF group were detected in 90% (19/21) of the sequenced isolates. Plasmids of the IncF type are one of the most common Inc types identified in humans and animals, especially in Asia, and most often carry AMR genes of the *bla* family (Rozwandowicz et al., 2018).

Long-read sequencing and hybrid assembly with Illumina short reads allowed more in-depth analysis of plasmids harboring beta-lactamases in our study. Hybrid assembly of the sequences resolved the correct plasmid replicons carrying *bla* genes, which would not have been possible with confidence from the fragmented short read data. As new assembly tools, such as Trycycler^3^ have been developed to resolve genomes from WGS data it is important to bear in mind the requirements for high-quality assemblies with these new tools, such as high enough coverage of the long reads. With the advancement and improved availability of long-read sequencing technologies plasmid analyses regarding AMR studies should prove to be even more affordable and accessible in the future.

Our analysis comparing plasmids carrying *bla* genes in this study with plasmid sequences deposited in open databases showed the plasmids in our study were similar with mostly livestock-derived plasmids obtained previously from various countries, but also with human clinical samples, especially from urinary tract infections. pZPK-A41.2-1 carried the P fimbriae encoding virulence gene papC which has been associated with uropathogenic *E. coli* infections (Yazdanpour et al., 2020). pZPK-A40.2-1 was also similar to a *mcr-1*-carrying plasmid isolated from a human urine sample from the United States (GenBank accession: KX276657.1), indicating a potential for IncF type plasmids being able to obtain new resistance genes. pZPK-C86.1-1_X1 was found the be a typical IncX1 plasmid in regards of carrying the PMQR gene *qnrS1*, which has been linked to *Salmonella* and *E. coli* from animal and human sources (Dobiasova and Dolejska, 2016). This plasmid was also found to be highly similar to a plasmid isolated from a human urinary catheter sample in Japan (GenBank accession: AP023429.1).

Five of the hybrid assembled plasmid sequences were obtained from raw broiler meat samples originating from the same batch number. Four of these carried the IncI-Iγ type plasmid, but all harbored a different *bla* gene, indicating diversity even among plasmids obtained from a homogenous origin. The similarity of all of the seven hybrid assembled plasmids, even from vegetable origin, to poultry and other livestock sources published previously also indicates certain plasmid-resistance gene combinations flourish in specific host environments.

Similarity to plasmids from small animal sources was also observed. pZPK-C61-1 of the IncI1-Iγ type with *bla*_CMY–2_ was highly similar to a plasmid obtained from a canine urinary tract infection sample in the UK (GenBank accession: CP023376.1). pZPK-C81.1-1 with *bla*_CTX–M–1_ also of IncI1-Iγ type was also similar to a *bla*_CTX–M–1_-carrying plasmid obtained from *E. coli* from a dog in Switzerland (GenBank accession: MG948334.1). The finding of similar transmissible plasmids from food products and small animals indicates a possible transmission route between food, animals and potentially humans who are in close contact with pets. This highlights the need for plasmid studies implementing a One Health approach, since horizontal gene transmission via plasmids does not know country or species borders.

Interestingly, long-read sequencing and hybrid assembly revealed chromosomally located beta-lactamases in two of the sequenced isolates. Long-read sequencing provides reliable information on the true location of resistance genes, as plasmid replicons and genes can be matched together. As plasmids have been recognized as successful drivers of AMR, more studies combining short- and long-read sequencing are needed in order to sequence plasmids as a whole (Valcek et al., 2019) and to gain a deeper knowledge of the multifactorial epidemiology behind the spread of AMR.

Interestingly, no ESBL/AmpC-producing Enterobacteriacea*e* were obtained from fruit or seafood samples in our study. It is noteworthy, however, that sample sizes were limited. In another study conducted in Spain, raw fish products and sushi were found to have a prevalence of ESBL-producing Enterobacteriaceae of 10.6 and 19.4%, respectively (Vitas et al., 2018). Local fish products in Vietnam have been found to have an ESBL-producing *E. coli* prevalence of 62.5% with a high level of multidrug resistance (Le et al., 2015). Other AMR bacteria have also been recovered from seafood products in previous studies (Boss et al., 2016; Ellis-Iversen et al., 2019). In our study, seafood samples consisted largely of frozen or precooked products, which may, together with limited sample size, explain the absence of ESBL producers. Fortunately, no cross contamination with ESBL/AmpC-producing Enterobacteriaceae was found in cooked meat products, as these products are often eaten without additional heating, posing a greater risk of transmission of bacteria. The finding is in line with an earlier study conducted in Spain (Vitas et al., 2018) but not in line with a study by Jiang et al. (2014), which found a 6.7% prevalence of ESBL producers in *E. coli* isolates recovered from cooked meat products in China. *bla*_CTX–M–1_, *bla*_CTX–M–9_, and *bla*_TEM–1_ were identified in the latter study, which may point to inadequate hygiene measures after heat processing or cross contamination, in which bacteria are introduced to products after heat processing. We did not recover any carbapenemase producing Enterobacteriaceae (CPE) in our study, although CPE have been recovered previously from meat, vegetable, and seafood products of Southeast Asian origin (Zurfluh et al., 2015a; Janecko et al., 2016; Sugawara et al., 2019). However, the selection with cefotaxime in the pre-enrichment of samples in our study might cause carbapenemase producing strains to be missed.

Earlier studies investigating the presence of ESBL/AmpC-producing bacteria in food products have partly differing results. A German study found only one out of 399 vegetable samples to be positive for cefotaxime-resistant *E. coli* (Kaesbohrer et al., 2019) but found cefotaxime-resistant *E. coli* in 74.9% of chicken meat and 40.1% of turkey meat. This is in line with our results, pointing to a higher prevalence of resistant Enterobacteriaceae in broiler meat and limited prevalence in vegetables. However, a Swiss study found 25.4% of vegetables imported from the Dominican Republic, India, Thailand, and Vietnam positive for ESBL-producing Enterobacteriaceae (Zurfluh et al., 2015b). This is remarkably higher than in our study with 5% of vegetable samples being positive for ESBL/AmpC-producing Enterobacteriaceae, although our study included samples from around the world, not only Asia and South America. In these continents, however, wastewater of human origin is commonly used for agriculture (Jiménez, 2006; Raschid-Sally and Jayakody, 2008). Using contaminated irrigation water may serve as a direct link to AMR contamination, especially if the water is applied to edible parts of fruits and vegetables (FAO and WHO, 2019). Antimicrobial-resistant bacteria have also been recovered from frozen products, vegetables, and ready-to-eat products from Chinese retail food (Ye et al., 2018). Ready-to-eat vegetables, in particular sprouts, have been previously recognized as a possible source of ESBL/AmpC-producing *E. coli* and *K. pneumoniae* (Kim et al., 2015).

Although food is not considered as the main source of human ESBL infections (Belmar Campos et al., 2014; Carmo et al., 2014; Börjesson et al., 2016; Day et al., 2019), similar ESBL strains have been occasionally found from human infections and foods (Irrgang et al., 2017; Yamaji et al., 2018; Day et al., 2019; Roer et al., 2019; Valcek et al., 2019). In a recent population-based modeling study, 18.9% of human ESBL/AmpC-*E. coli* carriage was attributed to a food source (Mughini-Gras et al., 2019), highlighting the importance of AMR surveillance and proper hygiene measures for food products. Our results also show highly similar plasmids have been previously identified from human samples, indicating a possible transmission route of ESBL-harboring plasmids via food sources.

Antimicrobial resistance is a global problem with an uneven distribution around the globe and an accumulated burden especially in low-income countries (Alsan et al., 2015; Seale et al., 2017; Hendriksen et al., 2019). Although the burden of AMR is not equal in all countries, globalization, traffic of people, animals, and food enables resistant bacteria to disseminate around the world. To track the spread of AMR, it is of vital importance to execute AMR surveillance programs and to study different food products from various food categories in order to detect changes in the distribution of resistant bacteria, genes, and plasmids. As plasmids have been recognized as important drivers of AMR, efforts should be put into developing rapid WGS pipelines able to accurately detect plasmids carrying antimicrobial resistance genes of high importance.

## Conclusion

Food products from different food categories around the world contain a wide variety of bacterial species resistant to third-generation cephalosporins among other critically important antimicrobials, with poultry meat serving as a rich reservoir of ESBL/AmpC-producing *E. coli* and *K. pneumoniae*. Although raw poultry meat was identified as the most common source of ESBL/AmpC-producing Enterobacteriaceae, the sporadic finding of resistant bacteria from fresh vegetable samples highlights the need for diverse One Health surveillance of AMR from multiple sources. Further studies should focus on identifying plasmids, resistance and virulence genes, and bacterial STs in food products relevant for human infections and food safety. The finding of high similarity between food-derived plasmids carrying *bla* genes with plasmids recovered previously from various sources globally highlights the risks posed by international trade, such as food products.

## Data Availability Statement

The datasets generated for this study can be found in online repositories. The names of the repository/repositories and accession number(s) can be found in the article/Supplementary Material.

## Author Contributions

PK, AH, MA, SN, EV, and A-LM contributed to the concept and design of the study. PK collected samples. PK and SN contributed to bacterial laboratory analysis. BK, MB, PK, and AH analyzed whole genome sequence data. MB and PK performed long-read sequencing. BK, PK, and MB performed subsequent analysis. BK and PK made visualizations for plasmid comparisons. PK and AH drafted the manuscript. MB, MA, and SN revised the manuscript. All authors have read and approved the final draft of the manuscript.

## Conflict of Interest

The authors declare that the research was conducted in the absence of any commercial or financial relationships that could be construed as a potential conflict of interest.

## Footnotes

1 https://www.ebi.ac.uk/ena/data/view/PRJEB37779

2 https://pubmlst.org/

3 https://github.com/rrwick/Trycycler
